# Evaluating statistical characteristics of biomarker-guided trial designs

**DOI:** 10.1186/1745-6215-16-S2-O83

**Published:** 2015-11-16

**Authors:** Miranta Antoniou, Andrea Jorgensen, Ruwanthi Kolamunnage-Dona

**Affiliations:** 1 University of Liverpool, Liverpool, Merseyside, UK

## 

Stratified or personalized medicine is a rapidly growing area of research and has attracted much attention in recent years not only in the field of oncology but also in other diseases. The aim of stratified medicine is to tailor the treatment given to a patient according to one or more personal characteristics. These characteristics can be demographic such as age or gender, or biological such as a genetic or other biomarker. Prior to utilising a patient's biomarker information in clinical practice, it is necessary that their validity has been robustly tested in terms of analytical validity, clinical validity and clinical utility. A number of biomarker-guided clinical trial designs have been identified for testing a biomarker's clinical utility in a literature search we undertook in the MEDLINE database, restricted to publications within the last ten years. These designs are essential in testing the effectiveness of a biomarker-guided approach to treatment, however to our knowledge no formal comparison of the designs in terms of their advantages and disadvantages, particularly from a statistical viewpoint, has been undertaken so far. The objective of this study is to examine some of the statistical features (e.g. power) of such trial designs, including a critical appraisal and comparison across designs in terms of these features. To achieve this, we applied statistical simulation methods and here we report on our findings.

